# Treatment Beliefs Reflect Unmet Clinical Needs in Lysosomal Storage Diseases: An Opportunity for a Patient‐Centered Approach

**DOI:** 10.1002/jmd2.70003

**Published:** 2025-02-26

**Authors:** Eleonore M. Corazolla, Mirjam Langeveld, Marion M. M. G. Brands, Barbara Sjouke, Carla E. M. Hollak

**Affiliations:** ^1^ Endocrinology and Metabolism Amsterdam UMC Location University of Amsterdam Amsterdam the Netherlands; ^2^ Laboratory Genetic Metabolic Diseases Amsterdam UMC Location University of Amsterdam Amsterdam the Netherlands; ^3^ Inborn Errors of Metabolism Amsterdam Gastroenterology Endocrinology and Metabolism Amsterdam the Netherlands; ^4^ Department of Paediatrics, Division of Metabolic Diseases Amsterdam UMC, Location University of Amsterdam, Emma Children's Hospital Amsterdam the Netherlands; ^5^ Department of Internal Medicine Radboudumc Nijmegen the Netherlands

**Keywords:** adherence, beliefs about medicines, Fabry disease, Gaucher disease, lysosomal storage diseases, mucopolysaccharidosis III

## Abstract

Despite life‐long pharmacotherapy for many people affected by lysosomal storage diseases, no data are available on their beliefs about their treatments. Therapeutic options range from disease‐specific, with varying levels of effectiveness, to purely supportive. This spectrum is illustrated by the three diseases Gaucher disease type 1 (effective disease‐specific therapies), Fabry disease (disease‐specific therapies with variable effectiveness), and mucopolysaccharidosis type III A/B (supportive care only). Employing the Necessity‐Concerns Framework of the Beliefs in Medicine Questionnaire, we investigated intra‐ and intergroup variability in adults with Gaucher disease type 1, Fabry disease, and parents of children with mucopolysaccharidosis type III A/B. Participants rated *necessity* and *concern* items on a Likert scale, leading to categorization as *accepting*, *skeptical*, *indifferent*, or *ambivalent*. Self‐reported demographic, disease‐, and therapy‐related data were also obtained. Eighty‐one surveys were completed. Gaucher disease respondents (*n* = 15) were overwhelmingly categorized as *accepting* (high necessity, low concern). Female Fabry disease respondents (*n* = 43) were almost equally distributed over all categories except *accepting.* Male Fabry disease respondents (*n* = 16) were mostly *ambivalent* or *accepting*, indicating overall high necessity scores but varying concern. All mucopolysaccharidosis type III participants (*n* = 7) were categorized as *indifferent* (low necessity, low concern). The Beliefs in Medicine Questionnaire emerged as a valuable and feasibly employable tool for individual and group assessments in these populations. It reveals differences in beliefs aligned with current unmet medical needs. Expansion of this approach is warranted to optimize personalized counseling on therapeutic choices and to align drug development with the needs and beliefs of potential recipients.

**Summary**

- Beliefs about prescribed medicines vary among people with different lysosomal storage diseases and are aligned with current unmet medical needs, which should be integrated into individual counselling and into the development of novel therapies.

## 1 Introduction

The pursuit of accessible and effective therapies for lysosomal storage disorders (LSDs) has historically progressed at varying rates for each disease, leading to pronounced differences in available therapeutic options [1]. Currently, for some LSDs, multiple disease‐specific therapies of varying effectiveness exist (Table S1). In these cases, counseling of therapy options is based on the expected added value for the individual patient and patient preferences. For people affected by other LSDs, a lack of disease‐modifying therapies leaves supportive care as the only option (Table S1).

These differences in choices and effectiveness are exemplified by three LSDs: type 1 Gaucher disease (GD1), Fabry disease (FD), and mucopolysaccharidosis type III A/B (MPS III). GD1 (OMIM 230800) is the attenuated form of Gaucher disease without primary central nervous system involvement in which Glucosylceramidase Beta 1  deficiency leads to macrophage storage of glycosphingolipids mainly affecting the liver, spleen, and bone marrow. While a slightly reduced life expectancy has been reported for a historic cohort of GD1, with appropriate and timely initiation of treatment, quality of life and life expectancy are expected to be normal [2, 3, 4] (Table S1). FD (OMIM 301500) is an X‐linked disease mainly impacting the cardiovascular, renal, and nervous system [5]. The phenotype is highly dependent on variations of the affected gene encoding for alpha‐galactosidase A (GLA) and sex [5]. Generally, classical males are the most severely affected with the poorest prognosis, and nonclassical females are asymptomatic with potentially *GLA*‐variant‐modified overall cardiovascular risk [5, 6, 7]. Therapeutic effectiveness varies widely in FD [8] (Table S1). Finally, MPS III or Sanfillipo disease (OMIM 252900) is a neurometabolic disease caused by mutations in genes responsible for the degradation of heparan sulfate and associated with progressive decline in cognitive development at an early age, decreased quality of life, and premature death [9, 10, 11]. Currently, there is no disease‐specific therapy for MPS III; hence, treatment consists of supportive care (Table S1).

Depending on the severity of their disease, people with GD1, FD, and MPS III take medication, in most cases for the duration of their life following diagnosis. Despite this, there are no data on these individuals' beliefs about their medicines. These data are potentially relevant in three contexts: Firstly, to enhance therapy adherence: real‐world data suggest higher adherence in GD1 and FD than in other chronic diseases [12, 13, 14, 15, 16, 17]. However, strictly monitored adherence of the standard‐of‐care therapy in a clinical trial led to significant biochemical improvement in people with FD, suggesting relevant improvement of adherence is possible [18]. Secondly, to incorporate individuals' beliefs about their medicine into counseling during the shared decision‐making process when initiating, changing, or discontinuing therapies. Lastly, to enable the beliefs of (sub)groups with a specific disease to inform decision‐making in the development of novel therapies.

In this study, we surveyed the beliefs of people affected by three LSDs with different therapy options, namely GD1, FD, and parents of children affected by MPS III, regarding their current therapeutic intervention(s). We employed the Necessity‐Concerns Framework of the Beliefs in Medicine questionnaire, which strongly and reliably correlates with therapy adherence [19, 20]. We assessed the inter‐ and intragroup variability in participants' perception of the necessity of and concerns about their medicines, emphasizing the potential for individual and group‐level engagement by clinicians and in therapy development.

## 2 Materials and Methods

### 2.1 Study Design and Participants

Three subgroups of people diagnosed with LSDs were selected to represent different current and past experiences with LSD‐related medicines: adults with GD1, adults with FD (male and female, classical and nonclassical), and people severely affected by MPS III types A and B. Recruitment took place via the outpatient clinics of the Dutch national referral centre for all three LSDs, the Amsterdam UMC location AMC, where almost all people diagnosed with GD, FD, and MPS III in the Netherlands are under care. Participants were recruited for an online survey that included both a questionnaire on gene therapy as previously published [21] as well as the BMQ as discussed here. Both parents of people with MPS III were invited to participate as a proxy for their underage and/or cognitively impaired children. The following inclusion criteria were applied: formal diagnosis of one of the selected LSDs over one year prior to the study, sufficient fluency in Dutch, legal competence, and in the case of FD and GD1, a minimum of 18 years of age. Therapy status was not considered during recruitment.

### 2.2 Beliefs About Medicine Questionnaire

The Beliefs about Medicines Questionnaire (BMQ) measures respondents' attitudes toward medicines [20]. It consists of two parts, which can be used independently: the BMQ‐specific and BMQ‐general. In this study, the BMQ‐specific, the part on the respondents' own medicines, was employed. The questionnaire was distributed in its official Dutch translation, with minor alterations for the MPS III respondents to linguistically correct for the parents' perspective (i.e., “my child's medicine” instead of “my medicine”; in Dutch “de medicijnen van mijn kinderen”) (Table S2). The BMQ‐specific consists of two factors: the five‐item factor “*necessity*” measuring participants' beliefs about the necessity of taking their specific medicine (Cronbach's alpha 0.77), and the six‐item factor “*concern*” measuring participants' concerns about potential adverse consequences of taking their medicine (Cronbach's alpha 0.79). The sixth item of the “*concern*” factor (“my therapy gives me unpleasant side effects”) is optional and was included in this study. Respondents indicate their level of agreement on a 5‐point Likert scale ranging from “*strongly disagree*” (1) to “*strongly agree*” (5). Higher scores indicate stronger beliefs (i.e., higher perceived necessity or more concerns, respectively). Based on the combination of scores, respondents are categorized as one of four attitudinal groups: *acceptant* (score high on necessity subscale and low on concern subscale), *ambivalent* (score high on necessity and concern subscales), *skeptical* (score low on necessity subscale and high on concern subscale), and *indifferent* (score low on necessity and concern subscales). Cumulative (meaning the numerical sum of) necessity or concern scores can also be analyzed separately.

### 2.3 Data Collection and Analysis

Potential participants were approached via a letter including information on the study. Once a signed informed consent form was returned, participants received a personal hyperlink to the questionnaire via email. In the case of MPS III participants, two hyperlinks were sent to allow parents to participate separately. In addition to the BMQ, demographic, disease‐, and therapy‐related data were collected. Age was captured as brackets of decades for FD and GD, and as binary categories (over/under 18 years of age) for MPS III to minimize the risk of identification. Technical support was offered to all participants, and reminders were sent two to four weeks after sharing the hyperlink. Only complete questionnaires were included in the analysis. Data analysis and visualization was performed in RStudio 4.3.0. Pearson's Chi‐squared test was used to compare the variables shown in Table S3. *p* values were adjusted for multiple comparisons using the Benjamini–Hochberg (false discovery rate) procedure. A two‐way ANOVA was employed with the dependent variable either the *cumulative necessity* or the *cumulative concern score*, and the Akaike information criterion (AIC) was used to estimate whether the ANOVA model with the variables *disease group* and *therapy status* as independent or interacting variables was the best fit model.

### 2.4 Ethical Approval and Privacy

After reviewing the study protocol, the Medical Ethics Committee of the Amsterdam UMC location AMC waived the need for ethical approval (W20_380 # 20.425). Compliance with data protection regulations under the General Data Protection Regulation was assessed by a data protection impact assessment under the supervision of the privacy officer of the Amsterdam University Medical Centres. All participants consented in writing to participate in the study.

## 3 Results

### 3.1 Participants

**FIGURE 1:**
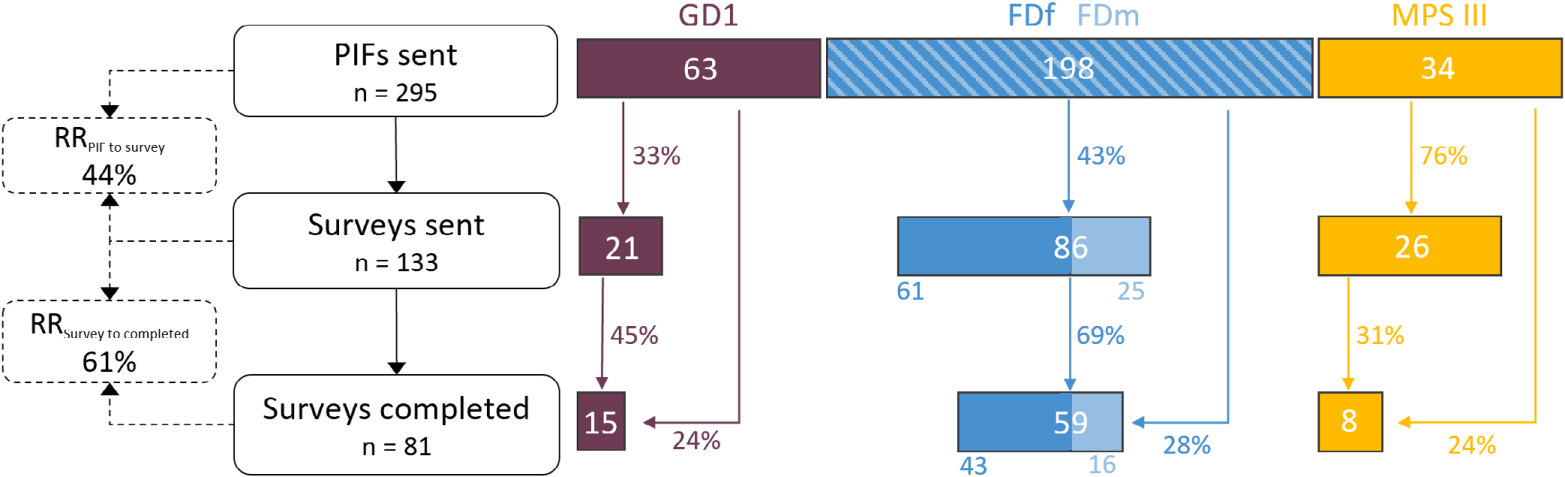
Recruitment flowchart. PIF: patient information folder; RR: response rate.

A total of 295 people (or their proxies) were approached (63 participants with GD1, 198 with FD, 34 with MPS III; Figure 1). After consenting to participation, the questionnaire was sent to 133 respondents (21 with GD, 86 with FD (61 women and 25 men), 26 with MPS III; Figure 1). Eighty‐one surveys were completed (15 with GD, 59 with FD (43 women and 16 men), seven surveys completed by eight caregivers representing nine people diagnosed with MPS III; Figure 1; Table 1). “The overall response rate (RR) was 44% of the approached people consented to participate (GD 33%, FD 43%, MPS III 76%) and 61% of participants who had consented completed the survey (GD 45%, FD 69%, MPS III 31%; Figure 1). This equates to 27% of all approached people completing the survey (GD 24%, FD 28%, MPS III 24%). No respondents identified as a gender other than “male” or “female.” Among GD1 participants, the age range and median were higher than among FD participants (median 65 years (range 50–79 years) vs. median 55 years (range 18–69 years), respectively; Table 1). All GD1 participants were receiving disease‐specific treatment at the time of the survey, mostly enzyme replacement therapy (ERT). In FD participants, 27 (46%) were not receiving disease‐specific treatment (five had discontinued treatment, 22 were therapy naïve; Table 1). Nobody with FD treated with chaperone therapy was included, as it is not currently reimbursed in the Netherlands. The seven MPS III surveys were completed by eight proxies (one survey was completed by two parents together) of nine people with MPS III, including two sets of two siblings each. Among the MPS III respondents, one survey (representing two affected people) stated having received disease‐specific therapy in the context of a clinical trial (Table 1).

**TABLE 1:** Demographic data of study participants.

	GD	FD women	FD men	MPS III^a^
Demographics				
Total (*n*)	15	43	16	9^b^
Female sex (*n* [% of total])	8 [53%]	43 [73%]		4 [44%]
Age				
< 18 years (*n* [% of total])	—	—	—	3 [33%]
> 18 years (*n* [% of total])	—	—	—	6 [67%]
18–29 years (*n* [% of total])	0 [0%]	7 [16%]	5 [31%]	—
30–39 years (*n* [% of total])	0 [0%]	5 [12%]	1 [6%]	—
40–49 years (*n* [% of total])	0 [0%]	7 [16%]	2 [13%]	—
50–59 years (*n* [% of total])	7 [47%]	14 [43%]	3 [19%]	—
60–69 years (*n* [% of total])	5 [33%]	10 [23%]	5 [31%]	—
70–79 years (*n* [% of total])	3 [20%]	0 [0%]	0 [0%]	—
Disease subtype				
Gaucher disease type 1 (*n* [% of total])	15 [100%]	—	—	—
Classical Fabry disease (*n* [% of total])	—	35 [81%]	9 [56%]	—
Nonclassical Fabry disease (*n* [% of total])	—	8 [19%]	7 [44%]	—
MPS III A (*n* [% of total])	—	—	—	5 [56%]
MPS III B (*n* [% of total])	—	—	—	4 [44%]
Current disease‐specific treatment^c^				
ERT (*n* [% of total])	13 [87%]	20 [47%]	10 [63%]	—
Imiglucerase (*n*)	12	—	—	—
Velaglucerase (*n*)	1	—	—	—
Agalsidase alfa (*n*)	—	1	0	—
Agalsidase beta (*n*)	—	19	10	—
SRT (*n* [% of total])	2 [13%]	0 [0%]	0 [0%]	—
Investigational treatment (clinical trial) (*n* [% of total])	0 [0%]	1 [2%]	1 [6%]	2 [22%]
Discontinued standard‐of‐care (*n* [% of total])	0 [0%]	4 [9%]	1 [6%]	0 [0%]
Treatment naive (*n* [% of total])	0 [0%]	18 [42%]	4 [3%]	7 [78%]

Abbreviations: ERT: enzyme replacement therapy; FD: Fabry disease; GD: Gaucher disease; MPS III: mucopolysaccharidosis type III; SRT: substrate reduction therapy.

^a^ MPS data pertains to the children represented by their proxies.

^b^ Seven questionnaires were completed by eight parents representing nine children with MPS III.

^c^ Use of non‐disease‐specific therapy was not explicitly asked in the survey and can therefore not be quantified.

### 3.2 BMQ Distribution per Disease Group

**FIGURE 2:**
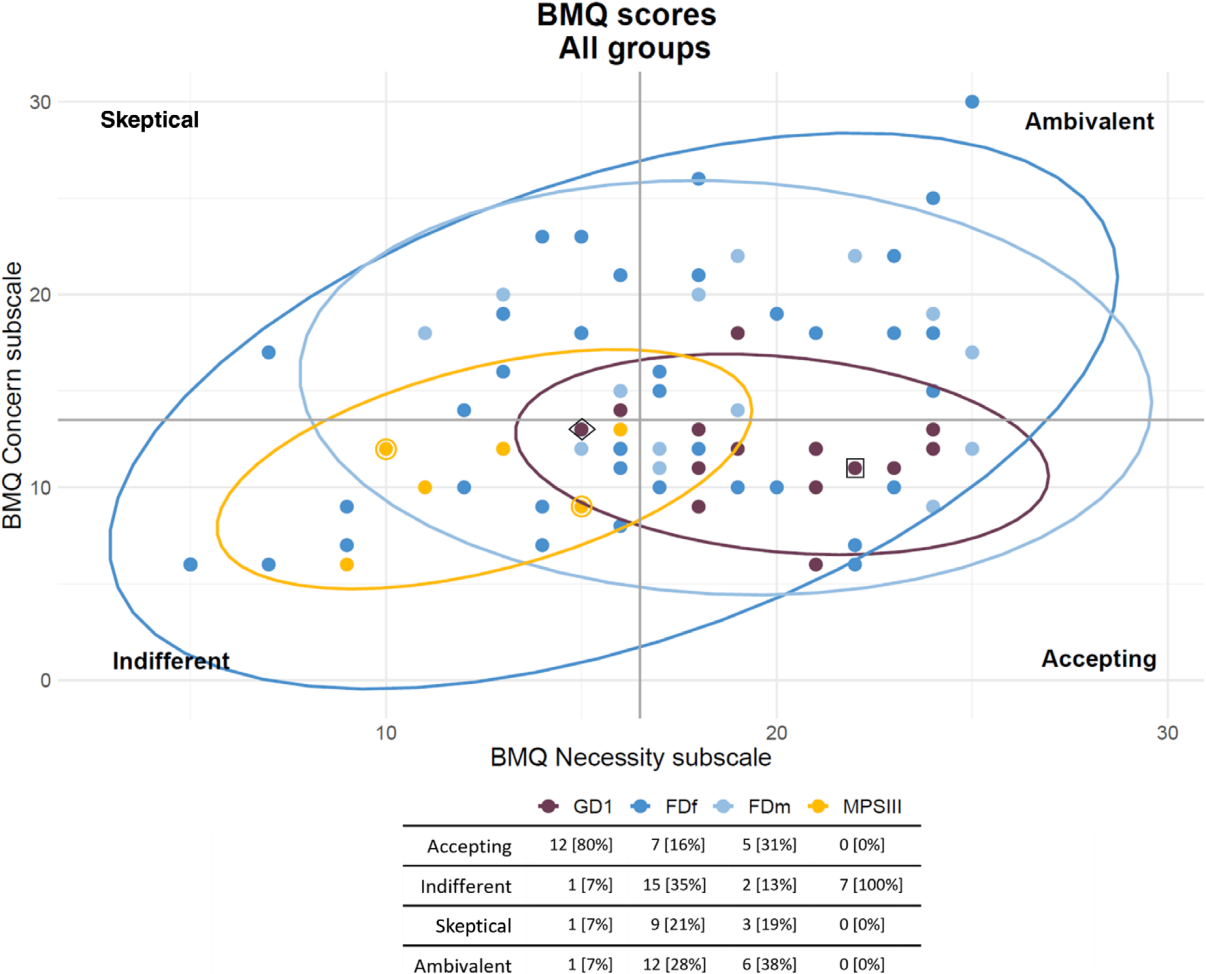
BMQ distribution per disease group. Respondents are categorized as one of four attitudinal groups: *Acceptant*—high score on the necessity subscale and low on the concern subscale, *ambivalent*—high score on the necessity and concern subscales, *skeptical*—low score on the necessity subscale and high on the concern subscale, and *indifferent*—low score on the necessity and concern subscales. MPS III numbers here represent the number of surveys, data points marked with an additional circle depict the outcome of one survey representing two people with the diagnosis. The dot inside a diamond is representative of three data points (not individually visible because of overlap): One GD, one FDf, one FDm. The dot inside a square is representative of two data points (not individually visible because of overlap): one GD, one FDm. FDf: women with Fabry disease; FDm: men with Fabry disease; GD1: Gaucher disease; MPS III: mucopolysaccharidosis type III.

Each disease group exhibited a different distribution of BMQ categories (Figure 2). The majority (80%) of GD1 respondents (*n* = 15) were “*accepting*” with only one respondent each scoring in the other three BMQ categories (Figure 2). In contrast, FD respondents were more evenly distributed over all four BMQ categories: female FD respondents (*n* = 45) were mostly “*indifferent*” (35%), followed by “*ambivalent*” (28%) and “*skeptical*” (21%) (Figure 2). Male FD respondents (*n* = 16) were mostly “*ambivalent*” (38%) followed by “*accepting*” (31%) (Figure 2). All MPS III respondents (*n* = 7) were “*indifferent*” (Figure 2). The correlation between disease groups and BMQ category was statistically significant (adjusted *p* value 4.02E‐5; Table S3).

### 3.3 Cumulative Necessity and Concern Scores per Disease Group

**FIGURE 3:**
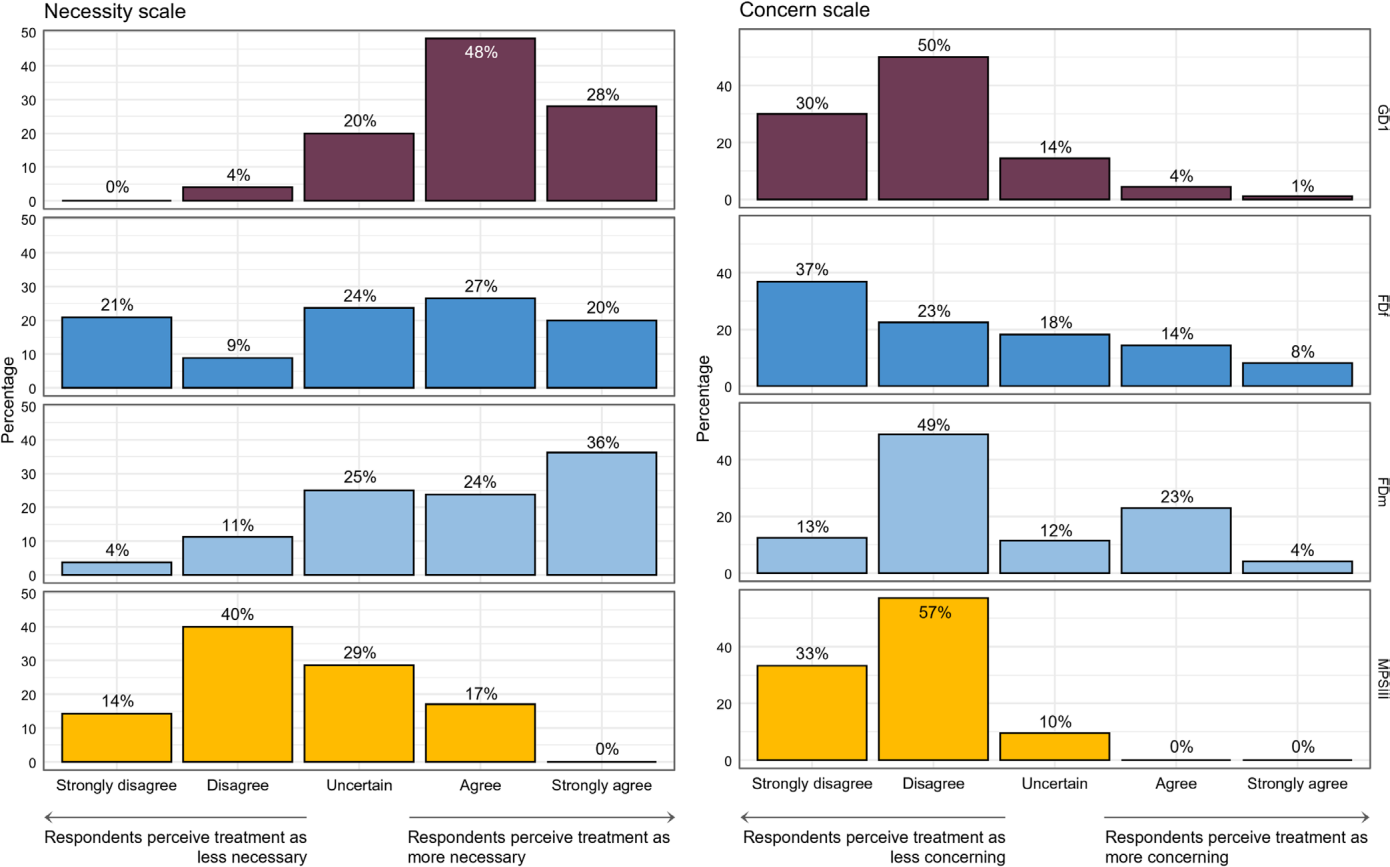
Percentage of responses to all cumulative items of each scale per disease group. The bar graphs depict the percentage of respondents in each disease group that chose each of the five options on a Likert scale on how strongly they (dis)agree with the items that comprise the necessity and concern scale. This illustrates differences between disease groups regarding the distribution of high and low necessity and concern scores. FDf: female respondents with Fabry disease; FDm: male respondents with Fabry disease; GD1: Gaucher disease type 1; MPSIII: mucopolysaccharidosis type 3.

Among the majority of GD1 respondents, cumulative necessity scores were high (76% (strongly) agree) while concern scores were low (80% (strongly) disagree) (Figure 3). Among female FD respondents, necessity scores were more evenly distributed than in other groups, with a skew toward high (47% (strongly) agree; 30% (strongly) disagree), and concern scores were low in the majority, but a larger fraction of respondents (strongly) agreed with concerns than in the GD1 group (22% FDf vs. 6% GD) (Figure 3). Among male FD respondents, high necessity scores and low concern scores were distributed similarly to those in GD1 (60% (strongly) agreed on the necessity score; 61% (strongly) disagreed on the concern score), however, there was a subgroup of 27% with high concern scores (Figure 3). In the MPS III group, the concern scores were low in a larger majority than in the other groups (90% (strongly) disagree), and, unlike all other groups, the necessity scores of the majority were also low (54% (strongly) disagree) (Figure 3).

### 3.4 Specific Items per Scale and Disease Group

Within the necessity scale, GD1 and FD participants agreed least with the item “without my medicines I would be very ill” (Figure S1). MPS III participants agreed most strongly with the item “my life would be impossible without my medicines” on the necessity scale (Figure S1). On the concern scale GD1 and female FD participants agreed similarly to all items, while male FD respondents agreed most strongly with “I sometimes worry about the long‐term effects of my medicines” and “I sometimes worry about becoming too dependent on my medicines” (Figure S1). MPS III respondents agreed least with “my medicines disrupt my life” on the concern scale (Figure S1).

### 3.5 Therapy Status as a Potential Confounder Between BMQ Category and Disease Group

*Therapy status* correlates with *disease groups* (adj *p* value 6.07^−21^), therefore, we analyzed whether it must be considered a confounding variable. In a combined analysis of all disease groups, *therapy status* correlated with BMQ categories: therapy‐naïve people were most likely to be categorized as *indifferent* and, to a lesser extent, *skeptic*, while people with ERT were most likely to be categorized as *acceptant* and *ambivalent* (adj *p* < 0.01; Table S3). *Therapy status* specifically correlated with *cumulative necessity scores* (adj *p* value 1.15^−5^) but not with *cumulative concern scores*. When analyzed as two independent variables using a two‐way ANOVA, *therapy status*, but not *disease group*, was significantly correlated with the *cumulative necessity score* (*p* < 0.01 for *therapy status*, *p* > 0.01 for *disease group*). Comparing this to a two‐way ANOVA in which *therapy status* and *disease group* were analyzed as interacting variables, the AIC analysis revealed that, in fact, the model in which no interaction between the variables *therapy status* and *disease group* is assumed fits best (AIC weight 0.91 meaning the model without assumption of interaction explains 91% of the total variation in the dependent variable *cumulative necessity score*; AIC without interaction 490 and with interaction 494). For the variable *cumulative concern score*, an identical analysis revealed no statistically significant correlation between *cumulative concern score* and *therapy status* or *disease group*. The correlation between *BMQ category* and *therapy status* was not statistically significant in any of the disease group or therapy status subsets when analyzed separately.

In summary, therapy status, not disease groups, is the strongest predictor of variation in the cumulative necessity scores of the BMQ in an independent, not interacting, manner. For the cumulative concern score, no such correlation is found. Subgroup analysis per disease group or therapy status is not possible.

### 3.6 Correlations Between BMQ Category and Other Factors

The disease groups were statistically significantly correlated with BMQ categories, specifically, GD1 was correlated with the category *acceptant*, FD (female) with *skeptic*, FD (male) with *ambivalent*, and MPS III with *indifferent* (adj *p* < 0.01; Table S3). Having experience with mild side effects was correlated with an *ambivalent* BMQ category, while not having that experience correlated with the category *indifferent* (adj *p* < 0.01; Table S3). Experience with clinical trial participation was correlated with an *acceptant* BMQ category, while absence of this experience correlated with *skeptic* and *indifferent* BMQ categories. Different age categories (for GD1 and FD only) were associated with different BMQ categories: necessity scores were low in the 30‐ to 49‐year‐old participants, and concern scores were low in the 40‐ to 59‐year‐old participants, resulting in an association of the category *ambivalent* with the youngest age bracket (18–30 years), followed by *skeptic/indifferent/ambivalent* (30–39 years), *indifferent* (40–49 years), *acceptant* (50–59 years), and *ambivalent* (60–69 years). Finally, the groups who had experience with severe side effects and for specifically, the GD1 group experience with additional medicine were statistically significantly correlated with BMQ categories, however, the groups who responded “yes” to those questions were very small (*n* = 3 and *n* = 1, respectively).

## 4 Discussion

In this study, the perceptions of people with different LSDs toward their medicine varied strongly between disease groups. People with GD1 agreed with the necessity of their medicine and had little concern about it. Women with FD responded very heterogeneously, with *accepting* as the smallest group. Men with FD overall agreed with the necessity of their medicine, but their concern varied. All MPS III respondents largely disagreed with the necessity of their children's medicines and were hardly concerned about it. The variation in BMQ necessity scores is almost completely explained by therapy status, not disease group, and these two variables are not confounders of each other. In summary, the BMQ categories and cumulative necessity and concern scores realistically represented the differences in how well medical needs are currently met for different LSDs.

The BMQ is a validated tool in adults with chronic diseases, including cohorts in the Netherlands, and has been widely used in research on parental/caregiver beliefs about the medicine of their children [20, 22, 23, 24, 25, 26, 27]. It is generally used as a counseling tool with the aim of improving pharmacotherapy adherence rates [19, 28, 29, 30]. In the few reports with real‐world data that are available for FD and GD, pharmacotherapy adherence rates are much higher than the usual 50% in other chronic diseases [31, 32, 33, 34], namely 92%–100% for ERT in GD1 and FD [12, 13, 14], 79% for substrate reduction therapy in GD1 [15], and 84%–92% for chaperone therapy in FD [16, 17]. Based on these data, the margin for improvement of adherence seems low. However, in a recent clinical trial in FD, many participants who had stabilized on enzyme therapy and continued the same treatment in the control arm experienced unexpected further biochemical improvement, probably as a result of better adherence [18]. This suggests that adherence may be lower than reported and its improvement can have relevant medical consequences. In the setting of this study in the Netherlands, adherence is not tied to reimbursement of medical costs, however, in other healthcare systems, improving access in this way may be an additional benefit of increasing adherence. Apart from improving adherence, counseling based on the BMQ might prevent (patient‐driven) abstention from treatment initiation or discontinuation of therapy. In the only report including data on therapy discontinuation, 20% (7/35) of people with FD chose to discontinue chaperone therapy, in some cases due to a lack of believing in its necessity [16]. We cannot estimate the potential for improvement on self‐chosen abstention from therapy initiation or discontinuation of therapies in GD1 or FD populations, since no data have been published on these aspects.

Adherence is not the only context in which the BMQ is potentially useful. We suggest its utility for LSDs in two additional contexts. Firstly, in individual counseling: the intra‐disease variability in necessity and concern scores among FD respondents in this study indicates the importance of integrating both disease‐specific and individual beliefs and concerns into counseling. In fact, a single session of individual counseling using the BMQ has successfully improved adherence in inflammatory bowel disease patients, proving that counseling actually improves adherence [35]. Further research on the impact of personalizing counseling (i.e., elaborating on the necessity of an individual's medication in their personal situation, or specifically asking about their concerns and discussing the evidence‐based validity of those concerns in their personal context) by utilizing a tool such as the BMQ on clinical and patient‐reported outcomes in LSDs is needed. Secondly, a context with potential for improvement is the development of novel therapies, which is undeniably necessary given the current unmet medical needs. As our groups' recent findings indicate, the risk profiles of some novel therapies in development for LSDs do not align with the risk tolerance of potential users, such as ex vivo gene therapy for GD1 [21]. To prevent this misalignment, we propose incorporating the preferences and beliefs of potential therapy recipients from the outset of the development of therapeutic interventions (i.e., evaluation of current therapy and attempting to reduce concerns and increase perceived necessity with novel therapies). Such an assessment would, for example, reveal the high skepticism of MPS III caregivers toward current therapy options we present in this study. Based on our previous work in this group, we believe this skepticism may be a sign of their desperation for therapies with a meaningful effect on the disease course, which is concomitant with a high risk tolerance for potential side effects of gene therapy as an example of a novel therapy, and thus highly relevant for therapy development [21, 36]. Additionally, employing this type of tool throughout (long‐term) clinical trials to compare beliefs regarding a novel therapy versus standard‐of‐care would allow for evaluation of whether participants' concerns are based on items related to drug administration (e.g., side effects, disrupting life) or to counseling‐related items (e.g., medicines are a mystery), thereby guiding efforts to shift patients' beliefs in their novel therapy in an attempt to enhance later real‐world adherence and satisfaction. The BMQ has proved in this study to be a useful and feasible tool to that end.

To our knowledge, this is the first study on perceptions of current therapy options among people with LSDs. Using a tool that measures beliefs on necessity and concern separately allows for a more nuanced interpretation of group‐level trends, for example, highlighting the subgroup of men with FD with high concern scores and the correlation between believing in the necessity of therapy and therapy status. A limitation of this study was the inability to correlate BMQ categories to characteristics within disease groups (e.g., classical and nonclassical FD) due to limited sample sizes. Additionally, the distribution of the BMQ in combination with a questionnaire on gene therapy may have introduced sampling bias, however, since demographic information was self‐reported within the survey, this is difficult to analyze. Based upon the only demographic data available on people who did not participate in the survey, namely disease group, it seems that while it varies per group which fraction of people discontinued the recruitment process at which step, ultimately the same fraction of people who were originally approached participated in the study in each disease group. The differences in disease group composition, specifically the lack of young adults with GD1 and the parental perspective as proxies in the MPS III group, may contribute to the intergroup differences. The generalizability of the specific BMQ outcomes for each disease to other international cohorts is limited as context‐specific influences beyond the scope of this study may contribute to beliefs in medicine of individuals and groups (i.e., historical and social contexts of medicine development, perception of the pharmaceutical industry). In this study, we did not ask participants to differentiate between their disease‐specific versus non‐disease‐specific medicines when completing the BMQ, therefore, we cannot evaluate the contribution of either type of medicine separately.

Nonetheless, this study yields two key findings: Firstly, the BMQ is valuable and feasible for assessments of patients' beliefs regarding their treatment in rare diseases, both at an individual and at a group level. Secondly, it reveals differences of beliefs in medicine between and within groups with different current therapy options and diseases aligned with unmet medical needs. We argue that tools such as the BMQ can and should be used to take an individual's preexisting beliefs into consideration during counseling on therapeutic options and to align novel therapy development with a nuanced view of patients' perspectives. This can be expanded to include children and teens [37, 38, 39]. We are currently discussing how to implement the BMQ into our clinical practice, specifically counseling of patients on therapy options in the outpatient clinic. A modified version of the BMQ could also be developed to allow research in other metabolic disease settings for example diets or the use of nutritional products. In essence, this is a translation of the “experience‐based codesign” technique from a clinical setting to the pharmacotherapy development pipeline [40, 41]. The BMQ can contribute to shifting healthcare for LSDs toward person‐centered care in which individual needs, preferences and values are prioritized and thus individuals' ownership of their healthcare decisions at multiple levels is promoted [42, 43].

## Author Contributions

The study was conceptualized by B.S. and E.M.C. The study design was discussed by an expert panel (M.M.M.G.B., M.L., C.E.M.H., and B.S.) and formalized by E.M.C. and B.S. Data were analyzed by E.M.C. E.M.C. drafted the manuscript together with C.E.M.H. All authors critically reviewed and approved the final version of the manuscript.

## Ethics Statement

The need for ethics approval was waived by the Medical Ethics Committee of the Amsterdam UMC, location AMC. All procedures were in accordance with the Helsinki Declaration of 1975, as revised in 2024.

## Consent

Participants provided written informed consent prior to receiving the questionnaire. Proof that informed consent was obtained is available upon request.

## Conflicts of Interest

Mirjam Langeveld and Carla E.M. Hollak were involved in pre‐marketing studies with Sanofi and Chiesi. Barbara Sjouke was involved in pre‐marketing studies with Protalix, Chiesi, Sanofi‐Genzyme, and Reneo Pharmaceuticals, none of which were related to the content of this study. Eleonore M. Corazolla and Marion M.G. Brands declare no conflicts of interest.

## Supporting information


**Figure S1.** Percentage of participants scoring on the Likert scale stratified by question for each disease group. FDf: female respondents with Fabry disease; FDm: male respondents with Fabry disease; GD1: Gaucher disease type 1; MPSIII: mucopolysaccharidosis type III.

Table S1.
Table S2.
Table S3.

## Data Availability

The datasets used and/or analyzed during the current study are available from the corresponding author upon reasonable request.
