# Supplementary figures and images for: Treatment Beliefs Reflect Unmet Clinical Needs in Lysosomal Storage Diseases: An Opportunity for a Patient‐Centered Approach
Source: JIMD Rep. 2025 Feb 26;66(2):e70003. doi: 10.1002/jmd2.70003 (PMC11864875; doi:10.1002/jmd2.70003)

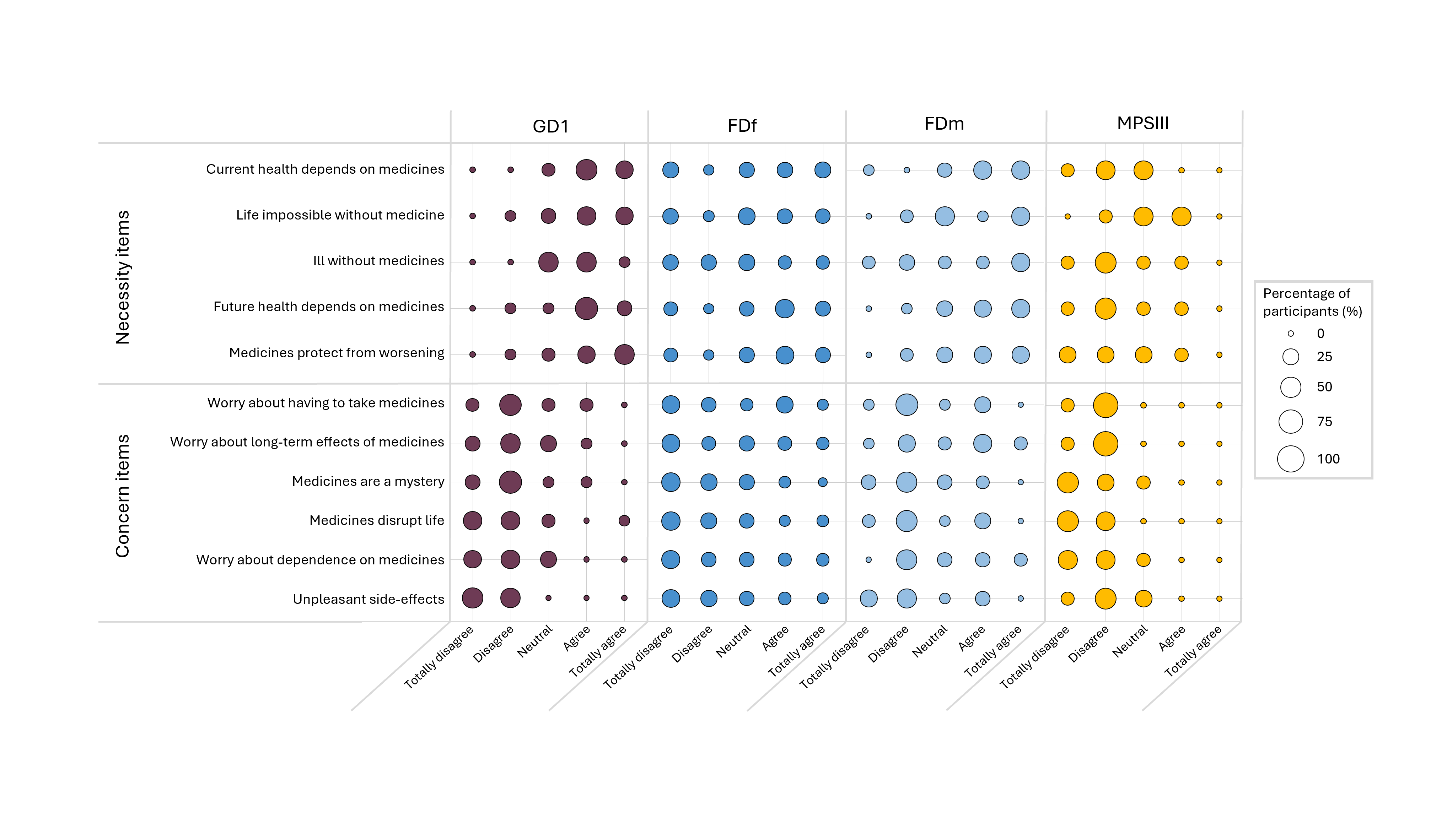

Supplement: Supplementary file 1 — Figure S1. Percentage of participants scoring on the Likert scale stratified by question for each disease group. FDf: female respondents with Fabry disease; FDm: male respondents with Fabry disease; GD1: Gaucher disease type 1; MPSIII: mucopolysaccharidosis type III. [file JMD2-66-e70003-s001.png]
