# Supplementary material for: Treatment Beliefs Reflect Unmet Clinical Needs in Lysosomal Storage Diseases: An Opportunity for a Patient‐Centered Approach
Source: JIMD Rep. 2025 Feb 26;66(2):e70003. doi: 10.1002/jmd2.70003 (PMC11864875; doi:10.1002/jmd2.70003)
Supplement: Supplementary file 2 — Table S1. Table S2. Table S3. [file JMD2-66-e70003-s002.docx]

**Supplemental Table 1: Summary of current non-experimental pharmacological disease-specific or non-disease-specific treatment options for GD1, FD and MPS III.** Based on typical per protocol use (in the case of GD1 and FD for adults only), individual adjustments are possible. In less severely affected individuals (e.g. mild type 1 GD, non-classical women with FD) disease-specific therapy may not be indicated at all.

*Taliglucerase alfa is registered for use in the USA, Israel, Brazil, and Canada only.

^#^ Agalsidase alfa is registered in the EU and elsewhere but not the USA.

Abbreviations: ACEi: Angiotensin converting enzyme inhibitor; ARB: angiotensin receptor blocker; CT: chaperone therapy; ERT: enzyme replacement therapy; FD: Fabry disease; GD: Gaucher disease; MPS III: Mucopolysaccharidosis type III; SRT: substrate reduction therapy

| **Therapy type** | | **Disease-specific pharmacological treatment options** | | | **Non-disease-specific (symptomatic) pharmacological treatment options** |
| --- | --- | --- | --- | --- | --- |
|  |  | **ERT** | **SRT** | **CT** |  |
| **Mechanism** | | *Recombinant form of missing/deficient enzyme that degrades substrate instead of endogenous enzyme* | *Substrate inhibitor: inhibits enzyme that synthesises substrate* | *Chaperone that stabilises protein folding, thereby preventing degradation and enhancing residual activity of deficient enzyme* |  |
| ***GD*** | Route of administration | Intravenous | Oral |  | Variable |
|  | Frequency of administration | Variable, usually every other week | 1-2 times daily |  | Variable |
|  | Effect on disease course | Highly effective at reducing visceral symptoms and preventing new visceral and skeletal symptoms. Less effective if initiated at very advanced disease stage (partial response, still at risk for complications). | Reduction of visceral symptoms, no evidence of superiority over ERT especially long-term |  | No effect on disease course |
|  | Side effects | Infusion reaction (rare), cough, headache, dizziness, GI symptoms, fatigue | Headache, dizziness, dysgeusia, palpitations, cough, GI symptoms, joint pain, fatigue |  | Variable |
|  | Additional information | Standard of care; high cost, burden of administration and necessity for cold chain may prevent access in some contexts | Registered for adults with GD1 who are normal, intermediate, or poor metabolizer of CYP2D6. Generally not advised as safe during pregnancy. |  | Commonly addresses (bone) pain, anaemia, thrombocytopenia, osteoporosis. |
|  | Drug names | Imiglucerase (Cerezyme®, Genzyme), Velaglucerase alfa (VPRIV®),  Taliglucerase alfa (Elelyso®/ Uplyso®)* | Eliglustat (Cerdelga®, Genzyme) |  | Variable |
|  | References | 44,45(p1),46–55 | 56–65 |  | 66 |
| ***FD*** | Route of administration | Intravenous |  | Oral | Variable |
|  | Frequency of administration | Every other week |  | Every other day | Variable |
|  | Effect on disease course | Attenuation of disease progression but new clinical events may still occur. Outcome in part dependent on timing of treatment initiation and disease phenotype. |  | Variable treatment responses, no valid comparison to ERT conducted; only relevant for people with amenable mutations | No effect on disease course |
|  | Side effects | Infusion reaction (dizziness, syncope; classical males), GI symptoms, fatigue |  | Headache, GI symptoms, fatigue, nasopharyngitis | Variable |
|  | Additional information | Standard of care; burden of administration and high cost and necessity for cold chain may prevent access in some contexts |  | Only effective in people with amenable mutations (35-50% of population with regional variations) | Commonly addresses acroparesthesias / neuropathic pain (e.g. carbamazepine), proteinuria (e.g. ACEi/ARB), arrythmias (anticoagulation, cardiac pacemaker/cardioverter) gastrointestinal symptoms |
|  | Drug names | Agalsidase alfa^#^ (Replagal®, Shire), agalsidase beta (Fabrazyme®, Genzyme) |  | Migalastat (Galafold®, Amicus) | Variable |
|  | References | 67–80 |  | 81–84 | 85 |
| ***MPS III*** | Route of administration |  |  |  | Variable |
|  | Frequency of administration |  |  |  | Variable |
|  | Effect on disease course |  |  |  | No effect on disease course |
|  | Side effects |  |  |  | Variable |
|  | Additional information |  |  |  | Commonly addresses pain, sleep disturbances, infections, epilepsy, excess oral secretions, diarrhoea, reflux. |
|  | Drug names |  |  |  | Variable |
|  | References |  |  |  | 86 |

**Supplemental Table 2: BMQ questionnaire in English (original) and official Dutch translation as used in this study.**

| Item number | Construct | Item in English (original) | Dutch translation |
| --- | --- | --- | --- |
| 1 | Necessity | My health, at present depends on my medicines | Op het moment hangt mijn gezondheid af van mijn medicijnen |
| 2 | Concern | Having to take medicines worries me | Ik maak me zorgen over het feit dat ik medicijnen moet nemen |
| 3 | Necessity | My life would be impossible without my medicines | Mijn leven zou erg moeilijk zijn zonder medicijnen |
| 4 | Concern | I sometimes worry about the long-term effects of my medicines | Soms maak ik me zorgen over de effecten die mijn medicijnen op de lange termijn kunnen hebben |
| 5 | Necessity | Without my medicines I would be very ill | Zonder mijn medicijnen zou ik heel ziek zijn |
| 6 | Concern | My medicines are a mystery to me | Ik ben onvoldoende op de hoogte van wat mijn medicijnen doen |
| 7 | Necessity | My health in the future will depend on my medicines | Mijn toekomstige gezondheid hangt af van mijn medicijnen |
| 8 | Concern | My medicines disrupt my life | Mijn medicijnen ontwrichten mijn leven |
| 9 | Concern | I sometimes worry about becoming too dependent on my medicines | Soms ben ik bang dat ik té afhankelijk zal worden van mijn medicijnen |
| 10 | Necessity | My medicines protect me from becoming worse | Mijn medicijnen voorkomen dat ik verder achteruit ga |
| 11 | Concern | My medicines have unpleasant side-effects | Deze medicijnen hebben onplezierige bijwerkingen |

**Supplemental Table 3: Levels of significance for the correlation between BMQ category and other variables**

MPS III could not be evaluated separately as all participants had the same BMQ category. Factors that were identical for an entire subgroup were not analysed (sex for FDm and FDf; experience with severe SE for FDm). Statistically significant results are marked as follows: * for p<0.05; ** for p<0.01.

Abbreviations: BMQ: beliefs in medicine questionnaire; ERT: enzyme replacement therapy; FDf: women with Fabry disease; FDm: men with Fabry disease; GD1: Gaucher disease; MPS III: mucopolysaccharidosis type III; SE: side effects; SRT: substrate reduction therapy

|  | **Independent variable** | **BMQ category (n)** | | | | | **Adjusted p-value** |
| --- | --- | --- | --- | --- | --- | --- | --- |
|  |  | **Sceptic** | **Indifferent** | **Acceptant** | **Ambivalent** | **Total** |  |
| Disease group | GD | 1 | 1 | 12 | 1 | 15 | 0.0000402** |
|  | FDf | 9 | 15 | 7 | 12 | 43 |  |
|  | FDm | 3 | 2 | 5 | 6 | 16 |  |
|  | MPS III | 0 | 7 | 0 | 0 | 7 |  |
| Therapy status | Naive | 6 | 17 | 1 | 4 | 28 | 0.003968** |
|  | ERT | 6 | 6 | 19 | 12 | 43 |  |
|  | SRT | 0 | 0 | 2 | 0 | 2 |  |
|  | Clinical Trial | 0 | 1 | 1 | 1 | 3 |  |
|  | Discontinued | 1 | 1 | 1 | 2 | 5 |  |
| Age category | <18 | 0 | 3^#^ | 0 | 0 | 0 | 0.0343467* |
|  | 18-29 | 2 | 3 | 2 | 5 | 12 |  |
|  | 30-39 | 2 | 2 | 0 | 2 | 6 |  |
|  | 40-49 | 2 | 4 | 2 | 1 | 9 |  |
|  | 50-59 | 4 | 5 | 12 | 3 | 24 |  |
|  | 60-69 | 2 | 4 | 6 | 8 | 20 |  |
|  | >69 | 1 | 0 | 2 | 0 | 3 |  |
| Sex | Female | 10 | 17^##^ | 13 | 13 | 53 | n.s. |
|  | Male | 3 | 8 | 11 | 6 | 28 |  |
| Experience with mild SE | Yes | 2 | 3 | 6 | 8 | 19 | 0.003968** |
|  | No | 11 | 15 | 18 | 11 | 55 |  |
|  | n/a | 0 | 7 | 0 | 0 | 7 |  |
| Experience for severe SE | Yes | 0 | 2 | 1 | 0 | 3 | 0.004584** |
|  | No | 13 | 16 | 23 | 19 | 71 |  |
|  | n/a | 0 | 7 | 0 | 0 | 7 |  |
| Experience with additional medication | Yes | 1 | 5 | 2 | 5 | 13 | n.s. |
|  | No | 12 | 20 | 22 | 14 | 68 |  |
| Experience with participation in clinical trial | Yes | 1 | 2 | 9 | 2 | 14 | 0.032384* |
|  | No | 12 | 23 | 15 | 17 | 67 |  |
| Total | | 13 | 25 | 24 | 19 | 81 | n/a |

^#^ For the indifferent category there are 3 MPS III participants under 18yo and 4 over 18yo but latter not added to table because rest of age bracket unknown

^##^ this refers to 17 forms in which female sex was reported, however two of these forms represent two female MPS III patients each making the total number of female participants in the indifferent category 19.
